# Magnetic Nanoparticle‐Based Upregulation of B‐Cell Lymphoma 2 Enhances Bone Regeneration

**DOI:** 10.5966/sctm.2016-0051

**Published:** 2016-08-02

**Authors:** Elizabeth Brett, Elizabeth R. Zielins, Anna Luan, Chin Chun Ooi, Siny Shailendra, David Atashroo, Siddarth Menon, Charles Blackshear, John Flacco, Natalina Quarto, Shan X. Wang, Michael T. Longaker, Derrick C. Wan

**Affiliations:** ^1^ Hagey Laboratory for Pediatric Regenerative Medicine, Division of Plastic Surgery, Department of Surgery, Stanford University School of Medicine, Stanford, California, USA; ^2^ Department of Material Science Engineering, Stanford University, Stanford, California, USA; ^3^ Electrical Engineering, Stanford University, Stanford, California, USA; ^4^ Institute for Stem Cell Biology and Regenerative Medicine, Stanford University School of Medicine, Stanford, California, USA

**Keywords:** B‐cell lymphoma 2, Osteogenesis, Scaffold, Magnetofection

## Abstract

Clinical translation of cell‐based strategies for tissue regeneration remains challenging because survival of implanted cells within hostile, hypoxic wound environments is uncertain. Overexpression of B‐cell lymphoma 2 (Bcl‐2) has been shown to inhibit apoptosis in implanted cells. The present study describes an “off the shelf” prefabricated scaffold integrated with magnetic nanoparticles (MNPs) used to upregulate Bcl‐2 expression in implanted adipose‐derived stromal cells for bone regeneration. Iron oxide cores were sequentially coated with branched polyethyleneimine, minicircle plasmid encoding green fluorescent protein and Bcl‐2, and poly‐β‐amino ester. Through in vitro assays, increased osteogenic potential and biological resilience were demonstrated in the magnetofected group over control and nucleofected groups. Similarly, our in vivo calvarial defect study showed that magnetofection had an efficiency rate of 30%, which in turn resulted in significantly more healing compared with control group and nucleofected group. Our novel, prefabricated MNP‐integrated scaffold allows for in situ postimplant temporospatial control of cell transfection to augment bone regeneration. Stem Cells Translational Medicine *2017;6:151–160*

**Significance Statement**

The use of adipose‐derived stem cells as transplanted cells in wounded areas is desirable for their regenerative potential, but they are difficult to use owing to their fragility. Enhancing their survival in the context of a calvarial defect can be achieved by stimulating antiapoptotic protein expression in the cells themselves, through a plasmid expression vector. The present study used a nonintegrating minicircle plasmid encoding B‐cell lymphoma 2 attached to a magnetic nanoparticle to facilitate in vivo transfection with temporospatial control (external magnetic field). This in situ system stimulates cell survival through gene expression and knock‐on bone regeneration through cell survival.

## Introduction

Craniofacial skeletal defects after trauma or tumor resection or resulting from congenital anomalies often present a significant reconstructive challenge. The limitations in the current treatment options have highlighted the need for the development of novel approaches with the ability to dramatically improve tissue repair. Cell‐based strategies to treat large craniofacial skeletal defects have shown promise; however, the survival of the implanted cells remains uncertain. Within the hypoxic/ischemic wound environment, the viability of transplanted progenitor cells can be dramatically compromised 1 2 3. Upregulation of the antiapoptotic factor B‐cell lymphoma 2 (Bcl‐2) within the transplanted cells themselves can enhance survival 4. The Bcl‐2 family of proteins regulates the “mitochondrial” pathway to apoptosis by preventing cytochrome‐C release from the mitochondria, which inhibits caspase activity 5, 6. Inhibition of apoptosis through enhanced expression of Bcl‐2, therefore, represents an attractive approach to augmenting cell survival for bone regeneration by implanted cells.

Viral transduction has been successfully used to upregulate Bcl‐2 in human adipose‐derived stromal cells (ASCs) to accelerate bone regeneration and wound healing 4. As an alternative, nonintegrating minicircle plasmids, a nonviral, episomal, covalently closed circular gene expression vector, have also been used for transient upregulation of Bcl‐2 gene expression 7, 8. As the minicircle plasmid lacks an origin of replication, it avoids the concerns raised regarding other integrating and/or viral vectors for gene therapy. In vivo, upregulation of Bcl‐2 using minicircle plasmids via nucleofection has been previously shown to enhance the survival of ASCs and increase bone formation in a critical‐size mouse calvarial defect 4.

In the context of this same mouse parietal bone defect model, we have generated a novel in situ technique to overexpress Bcl‐2 in transplanted human ASCs (hASCs), a multipotent cell population abundant in adipose tissue and capable of osteogenic differentiation. Magnetofection is the term given to strategically introducing DNA into cells using coated magnetic nanoparticles, coupled with the influence of an external magnetic field 9. Using a minicircle plasmid encoding human Bcl‐2 and green fluorescent protein (GFP) complexed to a specific magnetic nanoparticle (MNP), we have demonstrated the ability to transfect human ASCs in vivo.

Layers for nanoparticles were chosen based on function and biocompatibility. Iron oxide nanoparticles are an ideal vehicle, given their nontoxicity and degradability 10; branched polyethyleneimine (PEI‐B) has been shown to be an effective carrier of genomic material 11; and poly‐β‐amino ester (PBAE), a hydrolytically biodegradable polymer, has high transfection efficacy specifically with ASCs 12, 13. To facilitate in situ transfection, nanoparticle‐complexed Bcl‐2/GFP minicircles were incorporated into a hydroxyapatite‐coated poly(lactic‐coglycolic acid) (HA‐PLGA) scaffold 4, 14 onto which freshly harvested ASCs could be seeded. The present study investigated the effects of minicircle‐mediated Bcl‐2 upregulation using magnetofection on osteogenic differentiation and using our novel prefabricated scaffold to transfect ASCs after implantation in a temporospatially controlled manner to promote bone regeneration.

## Materials and Methods

### Magnetic Nanoparticle Synthesis

Particles were constructed using an iron oxide core coated with PEI‐B (Sigma‐Aldrich, St. Louis, MO, http://www.sigmaaldrich.com) by suspension in a 0.01% solution. Iron oxide cores (Sigma‐Aldrich) were coated using vortex agitation at 3,200 RPM in PEI‐B by dropwise addition of 20 μl at a time. The minicircles were then complexed by adding a 1 μg/μl solution of DNA. Finally, magnetic pull‐down was used to isolate the particles, followed by resuspension in a 0.01% PBAE solution in 25 mM sodium acetate to encapsulate the entire structure. (PBAE was provided by the laboratory of Dr. Fan Yang at Stanford University.) At each stage of the layering process, ζ potential analyzed particle charge (Malvern Zetasizer Nano‐ZS; Malvern Instruments, Worcestershire, U.K., http://www.malvern.com) and particle size were confirmed using dynamic light scattering (Brookhaven ZetaPALS; Brookhaven Instruments Corporation, Holtsville, NY, http://bic.com), with the assistance of the Center for Magnetic Nanotechnology at Stanford University.

### Minicircle Plasmid Synthesis

A human ubiquitin C promoter‐driven Bcl‐2 and GFP‐expressing minicircle was created from a pMC vector (CMV‐GFP‐T2A‐Luciferase Minicircle Parental Plasmid, BLIV501‐MN1; System Biosciences, Palo Alto, CA, http://www.systembio.com). In brief, restriction enzymes (XbaI and Age1; New England BioLabs, Ipswich, MA, http://www.neb.com) were used to ligate the parental plasmid, and primers for amplifying human Bcl‐2 inserts specific to the ligated parental plasmid were designed using the Clontech Primer Design Tool (Clontech Laboratories, Takara Bio, Mountain View, CA, http://www.clontech.com) ( supplemental online data). Polymerase chain reaction (PCR) was used to amplify the insert of interest, and the insert was cloned into ligated plasmid using a Cold Fusion Cloning Kit (System Biosciences, Palo Alto, CA, http://www.systembio.com). The plasmid was then used to transform ZYCY10P32S2T *Escherichia coli,* which were then grown and cultured in the presence of 0.25 μg/l selector antibiotic kanamycin. In brief, after overnight liquid culture growth in Terrific Broth (Thermo Fisher Scientific Life Sciences, Waltham, MA, http://www.thermofisher.com) and 50 μg/ml kanamycin, minicircle induction medium (Luria‐Bertani Broth; Sigma‐Aldrich), 0.04 N NaOH, and 0.01% L‐arabinose (Sigma‐Aldrich) were added to double the culture volume. The minicircle was induced by culturing at 32°C and purified using HiSpeed MaxiPrep kits (Qiagen, Hilden, Germany, http://www.qiagen.com). After extraction from *E. coli*, minicircle plasmids were stored at −20°C until use.

### Human Adipose‐Derived Stromal Vascular Fraction Harvest

Human lipoaspirate specimens from the abdomen were obtained from five healthy female donors after informed consent and under Stanford institutional review board approval (protocol no. 2188). The donors were aged 35–50 years and had no medical comorbidities. The lipoaspirate was processed to obtain the stromal vascular fraction containing ASCs. In brief, the lipoaspirate was washed twice with phosphate‐buffered saline (PBS) and then digested using type II collagenase (Sigma‐Aldrich) in Medium 199 (Cellgro; Mediatech, Corning, Manassas, VA, http://www.cellgro.com) at 37°C under gentle agitation for 30 minutes. Digestion was neutralized with an equal volume of Dulbecco's modified Eagle's medium (DMEM) plus GlutaMAX (Thermo Fisher Scientific Life Sciences) supplemented with 10% fetal bovine serum and centrifuged at 1,500 rpm for 20 minutes. The supernatant was aspirated and the cell pellet resuspended in DMEM and then passed through a 100‐μm pore cell strainer (Corning, Corning, NY, http://www.corning.com) before being centrifuged again at 1,500 rpm for 15 minutes. The cell pellet was resuspended in red cell lysis buffer and centrifuged again at 1,500 rpm for 15 minutes, leaving a stromal vascular fraction pellet. The number of live stromal vascular fraction (SVF) cells were counted using trypan blue.

### Flow Cytometry for Isolation of ASCs

Freshly harvested SVF cells from lipoaspirate were resuspended in fluorescence‐activated cell sorting (FACS) buffer (1 × PBS, 2% fetal bovine serum, 1% P188, 1% Pen‐Strep; Thermo Fisher Scientific Life Sciences), and were stained with anti‐CD45, anti‐CD34, and anti‐CD31 antibodies (eBioscience, Inc., San Diego, CA, http://www.ebioscience.com). Flow cytometry was used to quantify the proportion of ASCs, defined as CD45−/CD34+/CD31− cells. The fluorochromes used were anti‐human CD45 (Pacific Blue, eBioscience), CD34 (fluorescein isothiocyanate [FITC]), and CD31 (APC‐cy7). ASCs were then sorted using FACSAria II (BD Biosciences, San Jose, CA, http://www.bdbiosciences.com) and a 100‐μm nozzle.

### Minicircle Plasmid Transfection In Vitro

Cells were plated at equal density (100,000 cells per well in six‐well plates; Corning) and cultured using normal growth medium (Dulbecco's modified Eagle's medium, 10% fetal bovine serum, 1% Primocin [InvivoGen, San Diego, CA, http://www.invivogen.com]) until 80% confluence. The cells were then nucleofected or magnetofected in triplicate. For nucleofection, first‐passage ASCs were trypsinized, neutralized with culture media, and centrifuged at 1,000 rpm for 5 minutes. The cells were counted using a hemocytometer, and suspension solution was divided such that each 1.5‐ml tube contained 2,000,000 ASCs. The cells were resuspended in nucleofection medium specialized for mesenchymal stem cells (Lonza, Walkersville, MD, http://www.lonza.com) with 3 μg of minicircle Bcl‐2 plasmid DNA and transfected using setting B‐16 on a Nucleofector (Lonza, Basel, Switzerland, http://www.lonza.com) in accordance with the manufacturer's protocol. The cells were replated at a density of 80,000 cells per well (12‐well plate) and grown overnight without antibiotic. For magnetofection, first‐passage cells were grown at a density of 80,000 cells per well of a 12‐well plate, and 0.001 g of MNPs was added to each well in normal culture medium. Fluorescent microscopy was used to assess GFP and cellular colocalization, using 4′,6‐diamidino‐2‐phenylindole as a nuclear stain, per the manufacturer's protocol (Sigma‐Aldrich).

### In Vitro Viability and Osteogenic Assays

At 48 hours after transfection, the cells were lifted and counted using the viability dye trypan blue (Sigma‐Aldrich), which quantitatively measured cell survival between the nucleofected and magnetofected cells (with dead cells taking up trypan blue and thus appearing blue under the light microscope). Next, the cellular viability of ASCs after magnetofection or nucleofection with our Bcl‐2/GFP minicircle was determined by culturing cells with 1 μM staurosporine for 6 hours. Cells were subsequently lifted and stained with MitoTracker Red (Thermo Fisher Scientific Life Sciences). FACS analysis was used to quantify mitochondrial respiration, with positive phycoerythrin staining, indicating living cells. For osteogenic differentiation, second‐passage cells from the two experimental groups were seeded at equal densities (100,000 cells per well) in six‐well culture plates. After attachment, the cells were grown to at least 80% confluence before being cultured in osteogenic differentiation medium (ODM) 15, 16. Alkaline phosphatase and alizarin red staining and quantification were performed at 7 and 14 days, respectively, as previously described 15, 16. RNA harvesting of the transfected groups occurred on days 1, 5, and 7 after transfection to assess osteogenic gene upregulation using quantitative real‐time polymerase chain reaction (qRT‐PCR).

### Gene Expression (Apoptotic and Osteogenic)

RNA for osteogenic gene expression assay was collected at days 1, 5, and 7, and RNA for anti/proapoptotic gene expression was collected 4 days after transfection. RNA for magnetofected and nucleofected cells was harvested using TRIzol (Thermo Fisher Scientific Life Sciences) processed using the RNeasy Mini Kit (Qiagen). Reverse transcription was performed using TaqMan Reverse Transcription Reagents (Thermo Fisher Scientific Life Sciences). An ABI Prism 7900HT Sequence Detection System (Applied Biosystems, Foster City, CA, http://www.appliedbiosystems.com) was used to perform qRT‐PCR with the Power SYBR Green PCR Master Mix (Applied Biosystems) as the reporter. qRT‐PCR analysis was conducted to detect the gene expression levels of proapoptotic factor *SMAC/DIABLO*, antiapoptotic factor *Bcl‐2*, and pro‐osteogenic genes *RUNX2*, osteopontin (*OPN*), and osteocalcin (*OCN*). The expression levels of all genes were normalized to glyceraldehyde‐3‐phosphate dehydrogenase expression values.

### Scaffold Preparation and Visualization

Hydroxyapatite‐coated poly(lactic‐coglycolic acid) (HA‐PLGA) scaffolds were created from 85/15 poly(lactic‐coglycolic acid) by solvent casting and a particulate leaching process, as previously described 17. Scaffolds (4 mm in size) were donated by Dr. Min Lee, University of California, Los Angeles. Minicircle plasmids complexed to nanoparticles were suspended in UltraPure water (Thermo Fisher Scientific Life Sciences). Next, 20 μl of this minicircle‐nanoparticle solution was applied to the base of each HA‐PLGA scaffold, such that each scaffold contained 10,000 ng of plasmid DNA. To visualize the construct, scanning electron microscopy was performed. The scaffolds were dehydrated with alcohol, followed by hexamethyldisilazane for 30 minutes, and then air‐dried before sputter coating with gold. Visualization was performed using a Zeiss Sigma field emission scanning electron microscope (Zeiss, Oberkochen, Germany, http://www.zeiss.com). The scaffolds intended for in vivo placement were prepared with the same MNP concentration, air‐dried, and stored acellularly at 4°C until ready for surgical placement.

### Calvarial Defect Model

Adult male nude Crl:NU‐*Foxn1^ν^* CD‐1 immunocompromised mice (Charles River Laboratories International, Inc., Hollister, CA, http://www.criver.com) were used under approval of the Stanford Administrative Panel of Laboratory Animal Care (protocol no. 9999). Each experimental group had a sample size of 7. The mice were anesthetized and prepared for sterile defect surgery. Calvarial defects 4 mm in diameter were made in the right parietal bone of each mouse using a 4‐mm circular knife at 40,000 rpm (NSK Z500; Brasseler USA, Savanah, GA, http://www.brasselerusa.com). The underlying dura mater was left intact after the bone disc was removed.

### In Vivo Magnetofection

Once the calvarial defects had been created, each pre‐prepared scaffold was placed into the defect such that the surface of the scaffold containing the MNPs was in contact with the dura mater. Each scaffold then received 200,000 freshly harvested ASCs in 20 μl of DMEM on the top, MNP‐free surface of the scaffold. The skin was sutured over the defect. Analyzing the effect of the magnet was performed through two groups. One group was exposed to an external magnetic field and one was not. A sterile 1.2‐Tesla magnet (OZ Biosciences, Marseille, France, http://www.ozbiosciences.com) was placed on the skin overlying the scaffold for 20 seconds. The magnet was subsequently removed, and mice were treated similarly for the duration of the study.

### Analyzing Transfection Efficiency In Vivo

At 4 days after surgery, three mice from each group were sacrificed, the scaffolds were explanted, and each scaffold was separately trypsinized of all cells. The scaffolds were neutralized using fully supplemented DMEM, and a cell pellet was collected and resuspended in FACS buffer. Cells were assayed for endogenous GFP expression to assess successful transfection of plasmid, using FACS Aria II. GFP was detected as Alexa Fluor 488.

### Micro‐Computed Tomography Evaluation of Calvarial Healing

Bone healing was measured over 8 weeks using micro‐computed tomography (micro‐CT) analysis. Mice (*n* = 3 per group) were scanned using an Inveon Multi‐Modality positron emission tomography/CT scanner (Siemens, Munich, Bavaria, http://www.siemens.com), as described previously 18, 19. After a baseline volume measurement at week 0, serial imaging was performed every 2 weeks for a total of 8 weeks. The images were reconstructed as a three‐dimensional surface using the MicroView 3D Image User and Analysis Tool (Parallax Innovations, Ilderton, ON, Canada, http://www.parallax-innovations.com) 18. The scans were quantified using ImageJ (NIH, Bethesda, MD, http://www.imagej.nih.gov).

### Histological Analysis of Mouse Calvaria

At 1 week after scaffold implantation, one mouse from each group was euthanized and skull harvested for histological analysis. The skulls were immediately fixed in 4% paraformaldehyde and then exposed to EDTA (Thermo Fisher Scientific Life Sciences) decalcification solution at pH 7.4 for approximately 4 weeks. Following sufficient decalcification, the skulls were dehydrated, embedded in paraffin, and sectioned. Bcl‐2 immunohistochemical staining was performed on the sections using the manufacturer's protocol (anti‐human Bcl‐2 [raised in goat], FITC‐conjugated goat anti‐rabbit; Abcam, Cambridge, U.K., http://www.abcam.com), evaluating the ASC production of Bcl‐2 after successful in vivo magnetofection. Fluorescent images were taken using a ×40 objective (Leica Microsystems, Wetzlar, Germany, http://www.leica-microsystems.com), and were stacked using ImageJ (NIH). At the end of the 8‐week period of CT analysis and scanning, all the remaining mice were sacrificed, and the skulls were harvested and prepared for histologic examination as before. The sections were then stained with Movat's pentachrome to assess bone regenerate at the interface between the scaffold and bone. Images were taken at ×20 objective at the interface between the scaffold and parietal bone. In addition, 10 random slides from the center of the original defect in each animal were also stained with aniline blue and imaged at ×20. ImageJ (NIH) was used to convert colored micrographs to binary, and pixel densitometry was performed across the 10 images from each mouse.

### Statistical Analysis

Data are presented as the mean ± SD. Analysis of variance was used for multiple group comparisons, and two‐tailed Student's *t* tests were used to perform direct comparisons between two groups, with corrections for multiple comparisons. A *p* value of <.05 was considered statistically significant. All statistical analyses were performed using GraphPad Prism software (GraphPad Software Inc., La Jolla, CA, http://www.graphpad.com).

## Results

### Magnetic Nanoparticle Synthesis and Characterization

**Figure 1:**
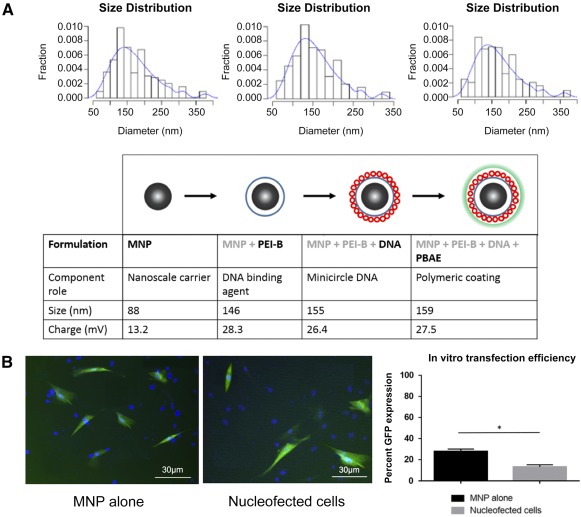
In vitro MNP formulation and cellular transfection. **(A):** Schematic of coating strategy of MNP using PEI‐B, DNA, and PBAE, respectively. The tabular data below the schematic show the role of each layer, average size calculated using dynamic light scattering, and ζ potential. The graphs above the schematic show dynamic light scattering measurements in distribution curves at each step of the layering process. **(B):** Fluorescent image of adipose‐derived stromal cells magnetofected and nucleofected with GFP‐expressing minicircle, showing transfection efficiency of both methods in vitro. Images were taken 48 hours after transfection; counterstaining was performed with 4′,6‐diamidino‐2‐phenylindole. Scale bars = 30 μm. Bar chart shows significant differences in GFP expression between groups (*, *p* < .05). Abbreviations: GFP, green fluorescent protein; MNP, magnetic nanoparticle; PBAE, poly‐β‐amino ester; PEI‐B, branched polyethyleneimine.

At each stage of the MNP layering process, the particle size and charge were measured (Fig. 1A). The iron oxide cores of 88 nm (per manufacturer) at 13.2 mV were coated in PEI‐B, leading to an increase in size and charge (155 nm and 28.3 mV). Subsequent addition of negatively charged minicircle plasmid DNA led to a decrease in particle charge (26.4 mV), followed finally by an external coating of positively charged PBAE. The final prepared particle had a size of 294 nm and a charge of 27.5 mV.

To evaluate the ability of MNPs to transfect ASCs relative to nucleofection with the same minicircle, fully layered MNPs were cultured with ASCs and were seen to have higher expression of GFP (31%) compared with cells nucleofected with the same GFP/Bcl‐2 plasmid (15.9%; Fig. 1B).

### Bcl‐2 Overexpression by Magnetofection Leads to Increased Viability Compared With Nucleofected Cells

**Figure 2:**
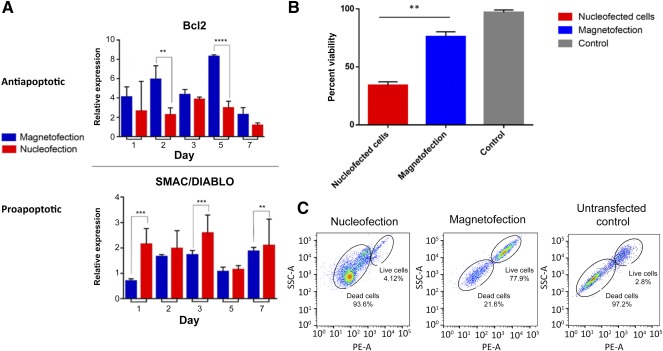
In vitro gene analyses and viability quantification. **(A):** Quantitative real‐time polymerase chain reaction analysis of pro‐ and antiapoptotic genes. Significantly greater Bcl‐2 expression (top graph) was noted in magnetofected cells at days 1, 2, and 5. In contrast, nucleofected cells showed significantly higher *SMAC/DIABLO* expression (bottom graph) at days 1, 3, and 7 compared with the magnetofected cells. Error bars show SD (**, *p* < .01; ***, *p* < .001; ****, *p* < .0001). **(B):** Graph depicting in vitro viability, quantified 48 hours after transfection using trypan blue staining. A significantly higher level of viable cells was present in the magnetofected cells (blue) compared with the nucleofected cells (red; **, *p* < .01). Error bars show SD. **(C):** Plots show fluorescence‐activated cell sorting analysis of cellular mitochondrial respiratory activity using MitoTracker Red (Thermo Fisher Scientific Life Sciences) staining after 6 hours of incubation with 1 μM staurosporine. Magnetofected cells were more resilient to staurosporine treatment (77.9% live cells) compared with nucleofected cells (4.12% live cells). Untransfected control shows nearly complete loss of cell viability (2.8% live cells).

The in vitro effects of minicircle‐driven Bcl‐2 overexpression using magnetofection or nucleofection were measured across several parameters. First, magnetofected cells showed significantly increased levels of Bcl‐2 transcript using qRT‐PCR compared with nucleofected cells (*p* < .01 and *p* < .0001) (Fig. 2A). Concomitant significant reduction in the proapoptotic gene *SMAC/DIABLO* was also observed, consistent with enhanced transfection of ASCs using magnetofection compared with nucleofection (*p* < .01 and *p* < .001) (Fig. 2A). Cell viability was detected 48 hours after transfection using trypan blue as a cellular viability dye. Magnetofected cells showed higher viability than nucleofected cells, 73% versus 49% (*p* < .05) (Fig. 2B). Finally, the cellular viability of ASCs after magnetofection or nucleofection with our Bcl‐2 minicircle was determined by culturing cells with 1 μM staurosporine for 6 hours. Subsequent cellular viability was evaluated by staining cells using MitoTracker Red (Thermo Fisher Scientific Life Sciences). We observed decreased mitochondrial activity in nucleofected ASCs, with only 4.12% live cells detected by FACS analysis compared with magnetofected ASCs, for which 77.9% of cells were viable and found to stain with MitoTracker Red (Fig. 2C). These data demonstrate that a greater number of ASCs remain viable after magnetofection with a Bcl‐2 expressing minicircle.

### Magnetofected ASCs Have Higher In Vitro Osteogenic Capacity Compared With Nucleofected Cells

**Figure 3:**
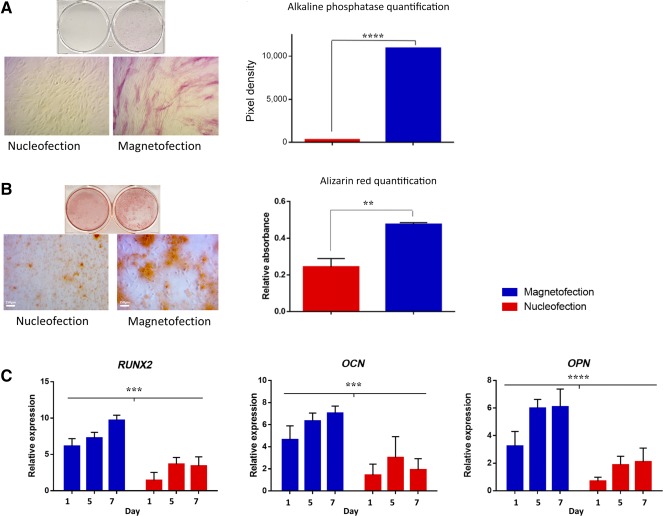
In vitro osteogenic capacity and gene expression. **(A):** Alkaline phosphatase staining performed on adipose‐derived stromal cells (ASCs) 7 days after transfection. Photographs of wells (top) and magnified (×10) images (bottom) show increased staining in magnetofected cells (right). Pixel densitometry revealed the difference in staining to be significant (****, *p* < .0001). **(B):** Alizarin red staining performed on ASCs 14 days after transfection. Photographs of wells (top) and magnified (×10) images (bottom) show increased staining in magnetofected cells (right). Optical absorbance quantification confirmed increased staining to be significant (**, *p* < .01). Scale bars = 150 µm. **(C):** Quantitative real‐time polymerase chain reaction showed differences in osteogenic gene expression of transfected cells cultured in osteogenic differentiation medium over 1 week. Nucleofected and magnetofected cells were analyzed at days 1, 5, and 7 for *RUNX2*, *OCN*, and *OPN*. Graphs show significantly upregulated osteogenic gene expression (***, *p <* .001; ****, *p* < .0001). Abbreviations: *OCN*, osteocalcin; *OPN*, osteopontin.

The ability of magnetofected and nucleofected ASCs to undergo osteogenic differentiation was subsequently evaluated by culturing cells in osteogenic differentiation medium for 7 days. Increased staining for alkaline phosphatase was observed among ASCs magnetofected with our Bcl‐2 expressing minicircle (Fig. 3A). Similarly, after 14 days of culture in osteogenic differentiation medium, alizarin red staining for extracellular matrix mineralization revealed enhanced bone differentiation among magnetofected cells compared with nucleofected cells (Fig. 3B). Parallel RNA harvesting from cells undergoing osteogenic differentiation showed significantly upregulated expression of osteogenic genes (*runx2*, osteocalcin, and osteopontin) in the magnetofected group at days 1, 5, and 7 of culture in ODM (Fig. 3C; *p* > .001 and *p* > .0001).

### Scaffold‐Based Magnetofection Is Dependent on an External Magnetic Field

**Figure 4:**
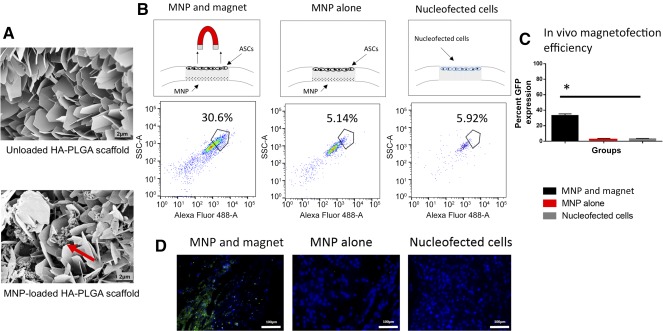
In vivo scaffold application and transfection efficiency. **(A):** Scanning electron microscopy images showing HA‐PLGA scaffolds. Top: Surface of an unloaded scaffold. Bottom: An MNP‐loaded scaffold. Red arrow indicates particles located on scaffold. Scale bars = 2 μm. **(B):** Schematic showing the in vivo scaffold strategies across three groups: MNP and magnet, MNP alone, and nucleofected cells (*n* = 3 per group). MNP and magnet group had the highest transfection efficiency (30.6%). Quantification of in vivo transfection efficiency was performed using fluorescence‐activated cell sorting (FACS) analysis. ASCs were harvested from scaffolds 48 hours after surgery and analyzed for GFP expression using FACS. **(C):** Quantification of in vivo transfection efficiency from FACS analysis across the three groups: MNP and magnet, MNP alone, and nucleofected cells (*, *p* < .05; *n* = 3 per group; error bars represent SD). **(D):** Immunohistochemical images showing presence of 4′,6‐diamidino‐2‐phenylindole/B‐cell lymphoma 2 (Bcl‐2) (fluorescein isothiocyanate labeled) in scaffolds harvested 1 week after surgery. MNP and magnet group micrograph qualitatively exhibited the most Bcl‐2 positive staining. Scale bars = 100 µm. Abbreviations: ASCs, adipose‐derived stromal cell; GFP, green fluorescent protein; HA‐PLGA, hydroxyapatite‐coated poly(lactic‐coglycolic acid); MNP, magnetic nanoparticle.

Having shown that in vitro magnetofection with our MNPs successfully upregulated Bcl‐2 expression and osteogenic differentiation, we developed a HA‐PLGA scaffold integrated with MNPs for in vivo cellular delivery, which was visualized using scanning electron microscopy (Fig. 4A). ASCs were seeded on top of this prefabricated scaffold and then placed into critical‐size mouse calvarial defects. The skin was closed, and the mice were subjected to a magnetic field. The scaffolds were then harvested after 2 days, and the cells were trypsinized off. As our nanoparticle‐complexed minicircle also contained a GFP reporter, the cells could be evaluated for postimplant transfection. Flow cytometry revealed 31% of cells retrieved off the scaffold to be GFP+ (Fig. 4B). Importantly, the cell‐scaffold‐MNP spatial orientation mattered, in that HA‐PLGA scaffolds loaded first with ASCs followed by application of the MNPs resulted in no detectable GFP+ cells ( supplemental online Fig. 1). Similarly, exposure to a magnetic field was critical, because prefabricated scaffolds loaded with ASCs but not treated with a magnet after implantation resulted in only 5% of cells transfected (Fig. 4B). Finally, as a comparison, ASCs were nucleofected ex vivo using the same minicircle and then loaded onto plain HA‐PLGA scaffolds. These constructs were then placed into critical‐size calvarial defects and removed after 2 days. With this approach, very few GFP+ cells could be retrieved from the scaffold. A similarly low percentage of viable transfected cells were noted as with our prefabricated scaffold without exposure to a magnetic field (Fig. 4B). Collectively, these findings demonstrate the ability of our prefabricated MNP scaffold to transfect cells after implant and that this ability is spatially dependent on magnetic field exposure of the cell‐scaffold construct. FACS analysis quantification showed a statistically significant increase in GFP expression in the MNP and magnet group (*p* > .05) (Fig. 4C). One week after implantation, the cell‐scaffold constructs were also explanted for histological analysis. Immunofluorescent staining of the samples revealed increased Bcl‐2 staining in the MNP and magnet group (18.75% MNP and magnet vs. 0.04% MNP and no magnet vs. 1.78% nucleofected cells; Fig. 4D).

### Enhancing the Survival of Transplanted Human ASCs in a Critical‐Size Calvarial Defect Increases Bone Healing

**Figure 5:**
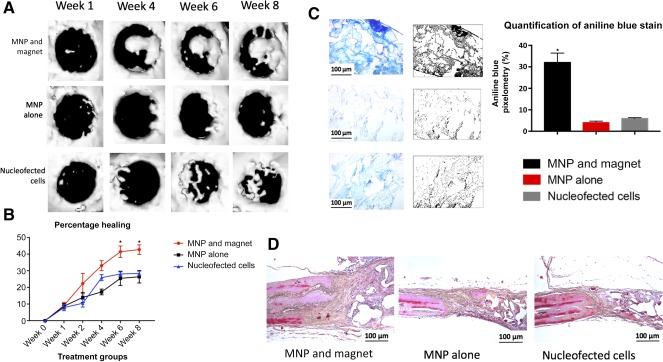
In vivo bone regeneration results and quantifications. **(A):** Image grid showing calvarial regeneration as measured by micro‐computed tomography of three groups across 8 weeks. The scans were reconstructed using MicroView (Parallax Innovations). **(B):** Quantification of calvarial healing. MNP and magnet group showed the highest bone regeneration among the groups, becoming significant at weeks 4 through 8. Error bars show SD (*, *p* < .05). **(C):** Representative histological images taken from the center of each scaffold harvested at week 8 using aniline blue staining and accompanied by their binary equivalent. Quantification of pixels in the adjacent graph revealed statistically significant differences in bone formation in the MNP and magnet group. Scale bars = 100 μm. Error bars show SD (*, *p* < .05). **(D):** Three histological images showing pentachrome staining of interface between parietal bone and scaffold harvested at week 8. MNP and magnet group exhibited the highest bone regenerate at the scaffold (yellow staining). Scale bars = 100 μm.

To determine whether in situ magnetofected ASCs could enhance bone formation, mice from each group were micro‐CT scanned every other week for 8 weeks, analyzing calvarial regeneration across the different treatments (Fig. 5A). The pixel densitometry of the CT scans numerically confirmed significantly greater regeneration noted at weeks 4, 6, and 8 in the MNP and magnet group (*p* < .05) (Fig. 5B). The bone regeneration with the scaffolds treated with nucleofected cells and scaffolds treated with MNP alone was found to be statistically equivalent at all time points. On harvesting at week 8, the scaffolds were prepared for histological sectioning and stained using aniline blue and Movat's pentachrome. The MNP and magnet group showed significantly increased bone regenerate in the center of the scaffold via aniline blue staining (Fig. 5C). Also, pentachrome staining revealed increased bone regenerate (yellow staining) at the interface between the scaffold and parietal bone (Fig. 5D).

## Discussion

Large, nonhealing bone defects represent a significant clinical challenge, and these are frequently seen in disease, trauma, and birth defects. Critical‐size bone injuries are often treated with distraction osteogenesis, autologous grafts, or synthetic polymeric materials, although none of these approaches are without limitations, and all can be associated with a variety of complications 20, 21.

The healing of critical‐size bone defects is dependent on bone resorption by osteoclasts and bone formation by osteoblasts. Increasing Bcl‐2 expression as a method of prohibiting apoptosis has been shown to actively regulate osteoblast and osteoclast differentiation, activity, and survival and, thus, bone remodeling 5, 22. Similarly, it was discovered using Bcl‐2 knockout mice that Bcl‐2 is indispensable for the anabolic activity of parathyroid hormone in bone, with wild‐type mice showing increased trabecular bone density after parathyroid hormone treatment compared with the Bcl‐2 knockout group 23. As such, selecting Bcl‐2 as a prosurvival gene in the context of healing bone is beneficial on several counts, including promoting cell survival, bone cell differentiation, and working in concert with endogenous hormones. Moreover, Bcl‐2 knockout mice exhibit disorganized collagen deposition in bone and differing osteoblast phenotype, suggesting a specific role for Bcl‐2 in extracellular matrix deposition 24. Enhanced Bcl‐2 expression has been previously shown to successfully augment cell‐based healing of bone defects and accelerate wound closure 4; however, to our knowledge, the introduction of a Bcl‐2 expression vector into post‐transplanted human cells in vivo has not yet been described.

B‐cell lymphoma 2 is a protein of oncogenic origin, showing multiple mechanisms of activation in cancer settings. In addition to changes to gene structure or copy number, hypomethylation of the gene and loss of endogenous microRNAs will also trigger Bcl‐2 expression, prolonging cell life and, thus, cell numbers 25. However, these methods of Bcl‐2 upregulation are characteristic of oncogenic cells and are distinct from transient artificial upregulation of Bcl‐2 such as through a nonintegrating, episomal expression vector or manipulating the expression pathway by blocking caspase‐3 26. The rationale behind choosing Bcl‐2 for prolonging ASC survival, therefore, represents harnessing a desirable characteristic from a cancer disease model. To ensure transient Bcl‐2 upregulation in the present study, we used a minicircle plasmid devoid of the parental portion containing an origin of replication. The resultant minicircle plasmid degradation profile has been thoroughly studied and understood since its inception, with previous data describing expression peaks at day 5, followed by a steady decline in expression thereafter 27, 28.

While maintaining the differentiation characteristics of pre‐adipocytes 29, hASCs have also been seen to commit to a neural lineage 30, endothelial‐like cells 31, and osteoblasts 32. Each of these three examples of differentiation entail different surface mechanical culture conditions, ranging from soft Matrigel (BD Biosciences) for endothelial cell differentiation to low amplitude/high frequency treatment for commitment to osteoblasts. In vitro, we confirmed the capacity of magnetofected hASCs to commit to an osteogenic lineage using osteogenic differentiation medium in culture. Although this is a common method for differentiating hASCs to osteoblasts, the in vivo portion of the present study involved the biomimetic mineral coating of hydroxyapatite on a PLGA scaffold, which has been seen to help lead hASCs down an osteogenic pathway 33. The combination of the osteogenic role played by Bcl‐2, the inductive nature of the hydroxyapatite‐coated scaffold, and with the prolonged survival of implanted cells thus sets the scene for enhanced bone regeneration.

Upregulating activity of Bcl‐2, a known oncogene, is a parameter that requires tight control. It is critical to minimize the off‐target effects of increasing Bcl‐2 expression in vivo, as any healthy cell population in a normal environment could potentially experience hyperplasia by proxy of Bcl‐2's antiapoptotic mechanism. However, if experienced by a target cell population, Bcl‐2 upregulation will work in favor of enhancing cell survival in the hostile, inflamed parietal defect environment. Cell exposure to MNPs was thus controlled by the use of an external magnetic field, which served to pull particles toward the targeted transplanted cells. Once the minicircle has been endocytosed by target cells, the plasmid will not integrate into nucleic DNA but, rather, will remain a self‐limiting expression vector 34. Of interest in the present study was the requirement of an external magnetic field to facilitate magnetofection. Once the MNP‐loaded scaffold was in situ with the overlying skin sutured, a magnet was placed over the site of the scaffold. Arranging the particles on the base of the scaffold (dural aspect) meant they were pulled upward through the ASC‐laden scaffold, toward the upper limit (dermal aspect), increasing the transfection of ASCs and providing enhanced in vivo regeneration. By strategically arranging the underlying layers of MNPs such that they will be dragged upward toward the overlying cells, a system is constructed in which physical magnetofection is reliably produced. Magnetofection in vivo involving an external magnetic field showed the highest rate of bone healing across all the groups on the micro‐CT scans (*p* > .05). This finding is in stark contrast to transplanted ASCs that had been nucleofected or ASCs that had not been exposed to a magnet in situ.

The concept of degradable, transient gene therapy has been explored in the present study in both the DNA carrier and gene vector itself. The aim of our clinical model was to enhance survival of transplanted cells in a translational way. By using a layered particle construct of nontoxic, degradable agents, combined with a nonintegrating expression vector, the strategy is such that the MNP or minicircle plasmid does not permanently remain.

## Conclusion

We have shown through magnetic‐assisted transfection of a Bcl‐2 expressing minicircle increased expression of the gene of interest, increased cellular proliferation, and decreased markers of apoptosis compared with nucleofected ASCs. Furthermore, our novel prefabricated MNP‐integrated scaffold allows for in situ postimplant temporospatial control of cell transfection to augment bone regeneration.

## Author Contributions

E.B.: conception and design, provision of study material or patients, collection and/or assembly of data, manuscript writing; E.R.Z. and A.L.: provision of study material or patients; C.C.O.: provision of study material or patients, collection and/or assembly of data, final approval of manuscript; S.S., S.M., and N.Q.: collection and/or assembly of data, data analysis and interpretation; D.A.: financial support, data analysis and interpretation; C.B. and J.F.: collection and/or assembly of data; S.X.W.: financial support, final approval of manuscript; M.T.L.: conception and design, financial support, administrative support, final approval of manuscript; D.C.W.: conception and design, financial support, administrative support, manuscript writing, final approval of manuscript.

## Disclosure of Potential Conflicts of Interest

The authors indicated no potential conflicts of interest.

## Supporting information

Supporting Information
