# Supplementary material for: Magnetic Nanoparticle‐Based Upregulation of B‐Cell Lymphoma 2 Enhances Bone Regeneration
Source: Stem Cells Transl Med. 2016 Aug 2;6(1):151–60. doi: 10.5966/sctm.2016-0051 (PMC5442739; doi:10.5966/sctm.2016-0051)
Supplement: Supplementary file 1 — Supporting Information [file SCT3-6-151-s001.pdf]

Supplemental Information – Wan et al.

Supplementary information:

Primers for vector and Bcl-2 insert

FORWARD: CATAGAAGATTCTATAATGGCGCACGCTGGGAGA

REVERSE: ATTTAAATTCGAATTCTCACTTGTGGCTCAGATAG

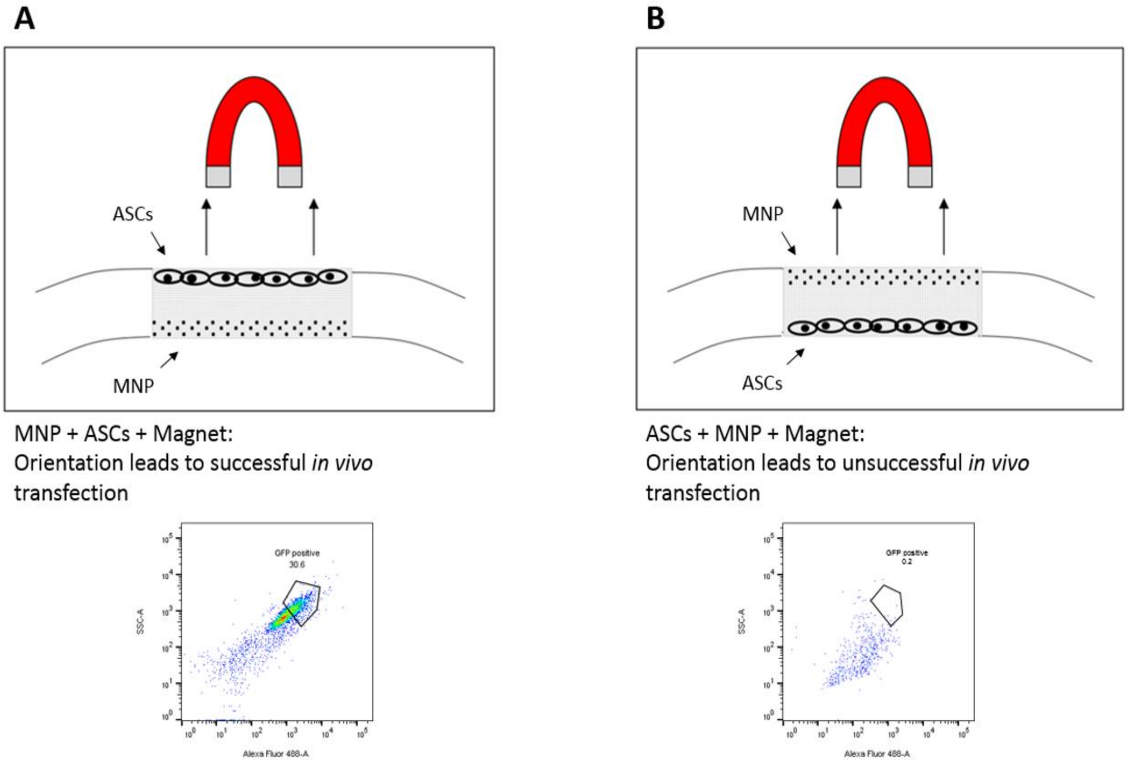

Figure S1:

A. Schematic showing MNPs at the base of the scaffold, ASCs overlying, and a magnetic field on the top, a stratification which results in successful magnetofection. FACS data shows GFP expression below. B) Schematic showing inverted orientation of ASCs and MNPs, a method in which the magnet drags the MNPs further away from the ASCs and therefore yielding a negative *in vivo* transfection. FACS data shows lack of GFP expression below.
